# Comment on “Characteristics and Risk Factors of* Helicobacter pylori* Associated Gastritis: A Prospective Cross-Sectional Study in Northeast Thailand”

**DOI:** 10.1155/2016/7654280

**Published:** 2016-08-09

**Authors:** Amin Talebi Bezmin Abadi

**Affiliations:** Department of Bacteriology, Faculty of Medical Sciences, Tarbiat Modares University, P.O. Box 14115-111, Tehran, Iran

We read with great interest the article by Tongtawee et al. concerning the analysis of 300 patients, who underwent esophagogastroduodenoscopy to investigate dyspeptic symptoms, who participated in this study from June 2014 to June 2015 [1]. However, some considerations we found can improve further research in current field. Infection with* Helicobacter pylori* (*H. pylori*) induces chronic inflammation in the human gastric microniche; moreover,* H. pylori*-related gastritis is highly linked with the development of gastric adenocarcinoma [2].

1. Genetic polymorphisms of* Mdm2 *SNIP309 are not valid enough to determine susceptibility of* H. pylori *infected individuals to the gastritis or even other gastroduodenal disorders. However, finding a significant association between a clinical disorder and a certain genotype requires a complex molecular investigation with considering various challenging elements.
2. Various types of gastritis described by Sydney standards should be mentioned; otherwise, data need to be recalculated. Thus, current question asked by authors requires more details to be answered.
3. The main limitation in this article is to analyze new biomarker in gastric cancer patients where this disease is not frequent. To examine and draw a better conclusion out of primary results by the authors, they need to investigate their results in a region with higher rate of gastric cancer.
